# TIDieR-telehealth: precision in reporting of telehealth interventions used in clinical trials - unique considerations for the Template for the Intervention Description and Replication (TIDieR) checklist

**DOI:** 10.1186/s12874-022-01640-7

**Published:** 2022-06-02

**Authors:** Daniel I. Rhon, Julie M. Fritz, Robert D. Kerns, Donald D. McGeary, Brian C. Coleman, Shawn Farrokhi, Diana J. Burgess, Christine M. Goertz, Stephanie L. Taylor, Tammy Hoffmann

**Affiliations:** 1 Primary Care Musculoskeletal Research Program, Brooke Army Medical Center, San Antonio, TX USA; 2 Department of Rehabilitation Medicine, School of Medicine, Uniformed Services University of Health Sciences, Bethesda, MD USA; 3 Department of Physical Therapy & Athletic Training, University of Utah, Salt Lake City, UT USA; 4 Departments of Psychiatry, Neurology, and Psychology, Yale University School of Medicine, New Haven, CT USA; 5 University of Texas Health, San Antonio, TX USA; 6 Pain Research, Informatics, Multimorbidities, and Education (PRIME) Center, VA Connecticut Healthcare System, West Haven, CT USA; 7 Yale Center for Medical Informatics, Yale University School of Medicine, New Haven, CT USA; 8 Department of Physical and Occupational Therapy, DoD-VA Extremity Trauma and Amputation Center of Excellence, Naval Medical Center, San Diego, CA USA; 9 VA HSR&D Center for Care Delivery and Outcomes Research, Minneapolis VA Medical Center and Professor, University of Minnesota Medical School, Minneapolis, MN USA; 10 Department of Orthopaedics, Duke University School of Medicine, and Core Faculty Member, Duke-Margolis Center for Health Policy, Durham, NC USA; 11 VA HSR&D Center for the Study of Healthcare Innovation, Implementation & Policy, VA Greater Los Angeles Healthcare System, Los Angeles, CA USA; 12 UCLA Departments of Medicine and Health Policy and Management, Los Angeles, CA USA; 13 Clinical Epidemiology, Institute for Evidence-Based Healthcare, Faculty of Health Sciences and Medicine, Bond University, Gold Coast, Australia

**Keywords:** Telehealth, Virtual care, Clinical trials, Reporting guidelines, Remote delivery

## Abstract

**Background:**

Recent international health events have led to an increased proliferation of remotely delivered health interventions. Even with the pandemic seemingly coming under control, the experiences of the past year have fueled a growth in ideas and technology for increasing the scope of remote care delivery. Unfortunately, clinicians and health systems will have difficulty with the adoption and implementation of these interventions if ongoing and future clinical trials fail to report necessary details about execution, platforms, and infrastructure related to these interventions. The purpose was to develop guidance for reporting of telehealth interventions.

**Methods:**

A working group from the US Pain Management Collaboratory developed guidance for complete reporting of telehealth interventions. The process went through 5-step process from conception to final checklist development with input for many stakeholders, to include all 11 primary investigators with trials in the Collaboratory.

**Results:**

An extension focused on unique considerations relevant to telehealth interventions was developed for the Template for the Intervention Description and Replication (TIDieR) checklist.

**Conclusion:**

The Telehealth Intervention guideline encourages use of the Template for the Intervention Description and Replication (TIDieR) checklist as a valuable tool (TIDieR-Telehealth) to improve the quality of research through a reporting guide of relevant interventions that will help maximize reproducibility and implementation.

## Key messages

- Remote delivery of health interventions is seeing exponential growth in both clinical practice and research.
- Poor replication of telehealth interventions from clinical trials will stagnate implementation and follow-on research.
- The TIDieR-Telehealth provides specific guidance for reporting details of telehealth interventions used in clinical research.
- Use of this checklist will improve the quality of research by maximizing the potential for reproducibility and implementation of telehealth interventions.

## Introduction

Recent global events revolving around the COVID-19 pandemic have led to a sharp rise in the remote delivery of healthcare. While remote delivery was used prior to the pandemic, COVID-19 became a catalyst forcing many healthcare institutions to pivot to remote delivery of care, especially for many non-urgent/non-emergent conditions where in-person appointments were more difficult to justify. This shift has been accompanied by an equal need to pivot delivery of many clinical trial interventions [1]. This was especially evident in trials studying management of acute, subacute, and chronic musculoskeletal pain where many nonpharmacological treatments (NPT) have relied on in-person visits, such as cognitive-behavioral therapies, physical therapy, and chiropractic care, among others. Even as the effects of the pandemic begin to ease, and in-person care begins to become more accessible again, this recent experience has highlighted many successes with the remote delivery of NPTs [2, 3]. It is likely that remote delivery will remain an option for providing NPTs, and clinical research employing remote intervention delivery methods will continue to proliferate. Unfortunately, health systems will have difficulty with the adoption and implementation of evidence-based telehealth interventions if ongoing and future clinical trials fail to report necessary details about their execution and delivery approach. Research that cannot be replicated leads to waste and setbacks in medical advancement [4].

Telehealth interventions within this context encompass any treatments that would traditionally be delivered in person but are now delivered remotely. It includes all the ways clinicians interact synchronously or asynchronously with patients using technology, including text messaging, videoconferencing, audio-only communication, mobile apps, and remote health monitoring [5]. It does not include tools or applications for self-management or ones that are void of any interaction with a healthcare provider or healthcare system.

### Telehealth - a new challenge to reproducibility of interventions

The Template for the Intervention Description and Replication (TIDieR) is a 12-item checklist developed to guide the thorough reporting of interventions with the goal of maximizing reproducibility [6]. The tool serves as an extension of the CONsolidated Standards for Reporting Trials (CONSORT) [7] checklist (Item 5) and the Standard Protocol Items: Recommendations for Interventional Trials **(**SPIRIT) [8] checklist (item 11), both for the planning and reporting of clinical trials. Without proper adherence to a reporting guideline like this, many interventions with proven effectiveness lack sufficient details for adequate replication in clinical practice or future research [9].

Poor reproducibility of interventions, in general, is a significant problem in the health care literature. In a recent review of systematic reviews, 87.9% of included studies had suboptimal adherence to reporting guidelines [9], and trials that utilized NPTs were among the most likely to fall into this category. The scope of NPT is vast and can be very nuanced, from exercise therapy to counseling or education to manual approaches. This complexity highlights why NPTs are more likely to suffer from poor replication and reinforces the importance of clear descriptions to maximize reproducibility. Yet, a large number of NPTs tested in clinical trials cannot be replicated [10–17]. With the growth of remote delivery options for NPTs, concerns about reproducibility will only magnify.

Recently, a joint initiative between the US National Institutes of Health (NIH), the US Department of Defense (DOD), and the US Department of Veterans Affairs (VA) was established to improve the management of pain and co-occurring medical and mental health conditions through the Pain Management Collaboratory [18]. The Collaboratory supports a significant investment in pain research through 11 large, multi-site pragmatic clinical trials that focus exclusively on delivery and evaluation of NPTs for pain. Pain is one of the leading causes of disability worldwide [19], and among the most common reasons to seek medical care [20, 21]. Pain conditions are one of the most commonly targeted by NPTs. The recent onset of the COVID-19 pandemic has brought many new and unique challenges to the delivery of interventions for trials in the PMC, requiring many to adapt and consider telehealth delivery [1, 22].

The COVID-19 pandemic has led to explosive growth in telehealth interventions [23, 24], and a great volume of research around these interventions is expected to follow. The original TIDieR checklist provides general guidelines for reporting NPTs but does not specifically address unique issues raised by the use of telehealth. Several TIDieR extensions have been developed for specific types of studies including placebo-controlled trials or studies examining policy interventions [25, 26]. The purpose of this paper is to provide practical considerations for adequately addressing the TIDieR checklist when reporting telehealth interventions in clinical trials. This paper provides recommendations and examples specific to telehealth interventions for each of the 12 original TIDieR checklist items.

## Development of the telehealth intervention guideline for the TIDieR (TIDieR-telehealth) checklist

### Workgroup infrastructure

**Fig. 1:**
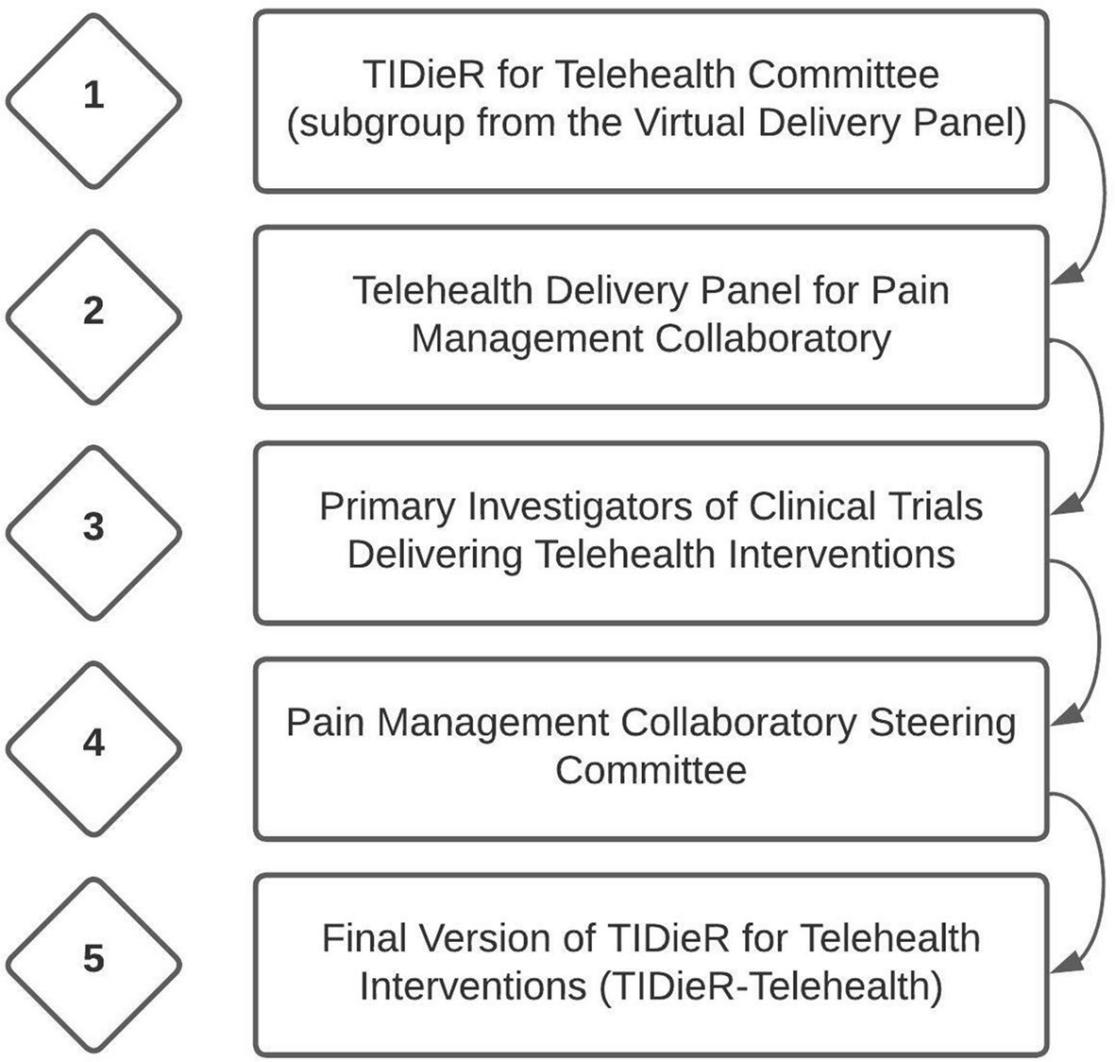
Steps in creation of TIDieR-VHI tool

The NIH-DOD-VA Pain Management Collaboratory is structured with a variety of internal workgroups to help meet the unique challenges of executing large-scale pragmatic trials. The Telehealth Care Panel workgroup within the Collaboratory was established to consider the challenges associated with the delivery of telehealth interventions. A small subgroup of members from the Telehealth Care Panel developed the first draft of the TIDieR-Telehealth checklist, which was then shared with the larger group for review, discussion, and added input through a series of group meetings. From there the Telehealth checklist was reviewed iteratively by 1) members of the Pain Management Collaboratory Implementation Science Workgroup, 2) the principal investigators of the pragmatic trials in the Collaboratory identified as delivering any part of their intervention remotely, and 3) all members of the Pain Management Collaboratory Steering Committee (Fig. 1). These individuals include diversity in gender, clinical setting (government, military, and civilian hospitals), and disciplines (psychologists, physical therapists, chiropractors, physicians, informaticists, sociologists, implementation scientists and public health experts). At each stage the checklist was revised and adapted based on feedback provided. Some trial investigators had originally planned for telehealth delivery, while others planned for in-person delivery but were required to pivot to telehealth delivery due to the current COVID-19 pandemic. Investigators were asked to evaluate each of their unique interventions across the checklist and provide feedback on the clarity, utility, and feasibility of addressing each item.

### Checklist items with telehealth considerations

A summary of each TIDieR item is provided in the next section, followed by a summary description of the item’s specific relevance to telehealth. Further details are presented in Table 1 which provides the TIDieR checklist adapted for Telehealth Intervention considerations, along with specific examples from the literature.

**Table 1:** TIDieR-Telehealth checklist

Original Item	Additional Considerations for Telehealth Interventions	Examples (Actual and Hypothetical)
BRIEF NAME		
1. Provide the name or a phrase that describes the intervention	Should include the word “**telehealth**” (or a term that very clearly indicates that an intervention is being delivered remotely - e.g., “telemedicine, **remote digital health, telephone**”) in the brief name that describes the intervention.	Telephone Coaching to Enhance a Home-Based Physical Activity Program for Knee Osteoarthritis: A Randomized Clinical Trial [27]. Telehealth Versus In-Person Acceptance and Commitment Therapy for Chronic Pain: A Randomized Noninferiority Trial [28].
WHY		
2. Describe any rationale, theory, or goal of the elements essential to the intervention	Provide the rationale for using a **telehealth** intervention. Is **remote** delivery used to expand access, or to enhance safety for participants? Is **remote** delivery an evidence-based option for the intervention? Is the goal to validate use of a traditional **in-person** intervention delivered in a **remote** format?	“Stigma and geographic barriers often prevent rural veterans from engaging in these evidence-based treatments. A large portion (37.7%) of VHA [Veterans Health Administration] enrollees diagnosed with PTSD live in rural areas. The objective of this pragmatic effectiveness trial was to test a collaborative care model designed to improve access to and engagement in evidence-based psychotherapy and pharmacotherapy for rural veterans [29].” “A 12-week standard smoking cessation program is available in Japan; however, it requires face-to-face clinic visits, which has been one of the key obstacles to completing the program, leading to a low smoking cessation success rate. Telemedicine using internet-based video counseling instead of regular clinic visits could address this obstacle [30].”
WHAT		
3. Materials: Describe any physical or informational materials used in the intervention, including those provided to participants or used in intervention delivery or in the training of intervention providers. Provide information on where the materials can be accessed (e.g. online appendix, URL).	What components does the **telehealth** intervention include (e.g., audio, video, name of platform or software)? What (if any) additional documentation, instruction and/or equipment was provided (loaned or given) to participants? Were participants that did not have access to the equipment or platform excluded?	“digital study materials (training protocol and video, PsychoPy experiment files and stimuli; (link), and analysis scripts (link) are publicly available in an Open Science Framework repository [31].” “At the end of the training sessions participants received a pair of prism goggles in a sealed opaque bag, a pointing sheet, written instructions, and a link to a video tutorial to take home [31].” “The details of the telemedicine system myIBDcoach have been described elsewhere (cite)(link). MyIBDcoach is a secured web page with an HTML application for tablet or smartphone. Exclusion criteria were an inability to read or understand the informed consent form, and lack of internet access by computer, tablet, or smartphone [32].” “Video counseling was delivered via Polycom PVX, a program installed on desktop computers and linked to the University study staff via the Internet. Each participating site received a desktop computer, webcam, and Polycom PVX software. A study technician installed equipment, tested connections with the site delivering the intervention, and trained clinic staff in equipment use and troubleshooting. The technician placed a binder with connection checklists, troubleshooting tips, and emergency phone numbers next to the study equipment. The technician also met with Internet service managers at each site to set up lines of communication for problem-solving connection issues that might arise throughout the trial [33].”
4. Procedures: Describe each of the procedures, activities, and/or processes used in the intervention, including any enabling or support activities.	Were the procedures for this intervention originally developed for in-person or **remote** delivery (number of minutes, open accessibility or requires sign-up/set-up)? What, if anything, was done to adapt procedures from in-person delivery?	“The study protocol consisted of a manualized, 8-week ACT for chronic pain intervention (see Intervention section) used in previous research (for non-virtual delivery). The in-person and virtual versions of Acceptance and Commitment Therapy (ACT) used a treatment protocol (manual available upon request) that was previously used in a randomized controlled trial (in person) comparing ACT with cognitive and behavior therapy for chronic pain (citation) modified for individual rather than group administration [28].”
WHO PROVIDED		
5. For each category of intervention provider (e.g. psychologist, nursing assistant), describe their expertise, background and any specific training given.	Who delivered the **telehealth** intervention? Was there any training that went into the delivery of the **intervention**? Who all was authorized/approved to deliver it and how did they achieve authorization approval (e.g., training, certification process)?	“The off-site telepsychologists delivered 12 sessions of individual CPT (veteran/military version) to interested patients. In addition to monitoring PTSD symptoms for the telepsychologist, the nurse care manager encouraged CPT initiation, attendance, and homework adherence. The off-site telepsychiatrist educated CBOC providers, supervised the TOP care team, and conducted interactive video psychiatric consultations as necessary [29].” “Once equipment was installed, the study project director conducted clinic staff training with each site via the Polycom system, in order to reinforce skills and build confidence in using the system. During this meeting, the project director reviewed study materials with the clinic staff, focusing on the clinic role in care such as reviewing prescription requests and providing medication prescriptions, as outlined below [33].” “participants were trained in person in how to carry out the treatment by a research psychologist [31].” “Study therapists were required to have graduate (at least master’s level) training in psychology. To avoid confounding the effects of mode of treatment with allegiance effects and therapist skill in nonspecific elements of therapy, study therapists conducted in-person as well as virtual delivery of treatment [28].”
HOW		
6. Describe the modes of delivery (e.g., face-to-face or by some other mechanism, such as internet or telephone) of the intervention and whether it was provided individually or in a group.	Indicate whether the intervention was delivered solely through **remote** methods or in a hybrid (**remote** + **in-person**) format. Synchronous versus asynchronous, unidirectional or bidirectional (could the participant/attendee ask questions, respond, interact and if so how - voice, chat, etc.?)	“Immediately after Research Session #2, participants were trained in person in how to carry out the treatment by a research psychologist according to a standardised protocol (available in study materials). Once the researcher was satisfied that the participant understood the treatment procedure, they performed the first treatment during this training session under the guidance of the researcher. After the training session, participants were instructed to perform twice-daily self-guided treatment sessions at home for two weeks [31].”
WHERE		
7. Describe the type(s) of location(s) where the intervention occurred, including any necessary infrastructure or relevant features.	Were clinicians in the clinic or at their home? Were patients in the clinic, another remote clinic, or at home?	“Because most computers were located in dedicated rooms in study clinics, participants could sign in at the clinic reception and go directly to the ... room for their session [33].” Three off-site PTSD care teams were located at the Veterans Affairs Medical Center (VAMC). Care manager and pharmacist activities were conducted by telephone (to the patient’s home). Psychotherapy and psychiatric consultations were delivered via interactive video (to the community-based outpatient clinic, from the VAMC). All feedback and treatment recommendations (from PTSD care team) were given to CBOC [Community Based Outpatient Clinic] providers via the electronic health record with requests for additional signatures when clinical action was needed [29]. Both interventions were home based. Both groups received a motivational app and remote supervision at home by a coach [34].
WHEN and HOW MUCH		
8. Describe the number of times the intervention was delivered and over what period of time including the number of sessions, their schedule, and their duration, intensity or dose.	Provide the planned intervention dosing (visits, frequency, duration, etc.) for the trial (expected treatment to meet optimal fidelity) and then also the number of actual visits received. Provide duration and frequency of sessions.	“In addition to the treatment they underwent during training (in person), participants were instructed to perform twice-daily self-guided treatment sessions at home for two weeks, resulting in 29 treatment sessions in total. They were instructed to commence the home-based treatment on the day following Research Session 2, perform one session in the morning and one in the evening, and record the start and end time of each session in a provided logbook [31].”
TAILORING		
9. If the intervention was planned to be personalized, titrated or adapted, then describe what, why, when, and how.	Describe the flexibility of the intervention to allow for any changes in or tailoring of the **telehealth intervention** for specific patients or groups.	“the PCPs could access the dermatologists online asynchronously via consultation or request a dermatologist to assume care (based on preference). Patients randomized to the online group had the option of accessing dermatologists online asynchronously [35].” Patients could choose their own device of preference (e.g., phone type, tablet, laptop computer) on which to receive the intervention.
MODIFICATIONS		
10. If the intervention was modified during the course of the study, describe the changes (what, why, when, and how).	If it was planned **remotely** or in-person and then had to be switched to the other, provide the timing, reasons, and rationale for the change.	The initial mindfulness intervention was planned for 8 sessions in person at the clinic. However, part-way through the trial (after treatment was completed for 87 [60%] patients), clinic closures resulted in a modification to the delivery, necessitating it to be delivered **remotely** for the remainder of the study.
HOW WELL		
11. Planned: If intervention adherence or fidelity was assessed, describe how and by whom, and if any strategies were used to maintain or improve fidelity, describe them.	Identify any specific strategies used to improve adherence to the **telehealth intervention**. Was there a plan to monitor and track fidelity of the **intervention?**?	“Therapist fidelity to Cognitive Processing Therapy (CPT) will be assessed via medical record review by dichotomously classifying each session as per protocol (ie, session 1, impact statement; sessions 2–7, stuck points; session 8, safety; session 9, trust; session 10, power/control; session 11, esteem and impact statement; and session 12, intimacy and impact statement). Overall CPT fidelity was defined as the percentage of sessions delivered per protocol [29].” To ensure treatment protocol adherence and competence in delivering treatment, therapists received 1 hour of weekly group supervision co-led by the second and senior authors [28]. “Patients’ training adherence was defined as a percentage counted from the total number of accomplished training sessions of an individual participant. Patients in the ITG group recorded the training sessions in the Polar Flow web application using the wrist heart rate monitor [36].”
12. Actual: If intervention adherence or fidelity was assessed, describe the extent to which the intervention was delivered as planned.	Did the **telehealth intervention** influence actual treatment adherence? Was the fidelity of the **telehealth intervention** reported?	“Approximately 20% (twenty-two) of the participants in the TELE group received, in addition to the telerehabilitation sessions, one or more face-to-face home visits (mean, 2.3 ± 2.2 visits). The documented reasons for visiting TELE group participants at home were a poor Internet connection or persisting technical problems (six visits), delayed technology installation (twelve visits), an abnormal profile of knee recovery (three visits), unavailability of clinicians (two visits), and anxiety of the participant (one visit). In addition, six participants did not receive the allocated intervention because of dissatisfaction with the result of randomization, a poor Internet connection, and a perception of a complete recovery [37].” “Among TOP patients attending any CPT sessions, 505 of the 514 sessions (98.2%) were conducted via interactive video, and the mean fidelity score to the CPT protocol was 79.8% [29].”

#### Item 1. Brief name: provide the name or a phrase that describes the intervention

The title and/or description of the intervention should make it clear the intervention is being delivered remotely and specify the telehealth modality used (e.g., telehealth, telemedicine, telephone-based, text-messaging). As noted earlier, telehealth interventions in this context would exclude any type of unsolicited, self-guided and unmonitored use of a tool or application that someone could access independently without requiring any interaction with a healthcare provider.

#### Item 2. WHY: describe any rationale, theory, or goal of the elements essential to the intervention

There should be a rationale specific to the decision to use the telehealth intervention which could include expanding the footprint of care, expanding the scope of care, convenience, improved adherence with the intervention, or the need to validate remote delivery of an intervention already proven effective when delivered in-person.

#### Item 3. WHAT (materials): describe any physical or informational materials used in the intervention, including those provided to participants or used in intervention delivery or in the training of intervention providers. Provide information on where the materials can be accessed (for example, online appendix, URL)

The specific tools and materials relevant to the delivery of the telehealth intervention should be described. For example, are specific devices or software necessary? Do providers or patients need any particular equipment and, if so, how are these materials accessed by providers (to deliver the intervention) or by patients (to receive the intervention)? When available and appropriate, these materials should be shared in a media that can be easily accessed (e.g., supplementary appendix, URL). Describe whether the platform being used is accessible to the public or has access restrictions (such as only to study participants).

#### Item 4. WHAT (procedures): describe each of the procedures, activities, and/or processes used in the intervention, including any enabling or support activities

Specific procedures relevant to remote delivery should be addressed. Does the patient need to create an account or require a specific code to access content? Are there any prerequisites or training procedures necessary prior to delivering or receiving the intervention remotely? If applicable, how were the procedures for delivering an in-person intervention adapted to accommodate remote delivery?

#### Item 5. WHO provided: for each category of intervention provider (e.g. psychologist, nursing assistant), describe their expertise, background, and any specific training given

Provide background on the training or credentials necessary to deliver the telehealth intervention. How do these requirements differ from those necessary for in-person delivery of the intervention?

#### Item 6. HOW: describe the modes of delivery (e.g., face-to-face, internet or telephone) of the intervention and whether it was provided individually or in a group

For this item, remote delivery may already be highlighted as the overall “how”, but additional clarification can be provided, to include whether the telehealth intervention was delivered individually or in a group, synchronously or asynchronously. Are there multiple options to the mode of delivery (i.e., patients could choose the option of receiving it voice only versus both video and voice)? If there is an asynchronous component, define what portions of the intervention are synchronous versus asynchronous and describe them accordingly. If there are both in-person and remote components to the intervention, adequately address details of each.

#### Item 7. WHERE: describe the type(s) of location(s) where the intervention occurred, including any necessary infrastructure or relevant features

This item has relevance for both patients receiving and clinicians delivering the telehealth intervention. For example, the intervention could come from a virtual medical center (e.g., vavmc.com). In this scenario a physical clinical structure could be established that clinicians travel to and operate from to deliver care remotely to patients at home. In contrast, there could be a physical location where patients travel (a satellite clinic or community-based outpatient clinic closer to their home) where the patient would receive care remotely from a specialist that would otherwise be located several hours or even days of travel away. If there is an asynchronous component, similar details should be provided about where that portion of the treatment would take place. If there are both in-person and remote components to the intervention, adequately address details of each.

#### Item 8. WHEN AND HOW MUCH: describe the number of times the intervention was delivered and over what period of time including the number of sessions, their schedule, and their duration, intensity, or dose

If there were a set number or range of remote visits, provide this information. If not, provide how the decision was made to end the intervention. Did the visits have to be completed within a certain timeframe? Was there a certain number of visits or treatment intensity (i.e., treatment dose) required to meet a minimum threshold of treatment fidelity? If any attempt was made to provide flexibility in the scheduling of appointments to accommodate the patient’s schedule (e.g., after work hours, evening) then mention that as well. If the intervention included an asynchronous component, differences in number, duration, intensity, frequency, and dose between the asynchronous and synchronous sessions should be included. If there are both in-person and remote components to the intervention, adequately address details of each.

#### Item 9. TAILORING: if the intervention was planned to be personalized, titrated, or adapted, then describe what, why, when, and how

Titration and modification of a general intervention can be based on a variety of parameters such as prior response to treatment, risk/prognosis stratification, or even personal preference. For a remote intervention, explain how and when these decisions were made, particularly within the constraints of a remote environment. Does the rationale to personalize, titrate and adapt the intervention differ in a remote compared to traditional setting? Describe how the personalization or adaptation will occur within remote settings. If there is an asynchronous component, provide details about how it was tailored or personalized. If there are in-person and remote components to the intervention, provide tailoring details of each.

#### Item 10. MODIFICATIONS: if the intervention was modified during the course of the study, describe the changes (what, why, when, and how)

Clarify how the study was originally determined or defined - as an intervention always planned for telehealth delivery, as an adaptation of a traditional intervention always planned to be delivered remotely, or as a telehealth intervention that was initially planned for in-person delivery but later changed to remote delivery based on methodological constraints or response to other study challenges? Make sure the rationale for the modification (i.e., technical constraints, implementation constraints) and the modification details relevant to the telehealth component of the intervention are clear. If there was an asynchronous component to the intervention, describe any modifications made to it, if any.

#### Item 11. HOW WELL (planned): if intervention adherence or fidelity was assessed, describe how and by whom, and if any strategies were used to maintain or improve fidelity, describe them

Assessment of treatment adherence is a challenge for intervention trials in general [38], and the problem can become amplified with telehealth interventions. Adherence can substantially alter the treatment effect [39], and therefore an important factor to consider when interpreting study results. Researchers should consider any unique components of adherence or fidelity associated with the remote delivery of the intervention. How was fidelity of the telehealth intervention monitored and how was adherence to the treatment measured? The same technology allowing for remote delivery of interventions can also provide robust and sophisticated methods for measuring treatment compliance. How do these surveillance methods differ for telehealth interventions compared to receiving the intervention in a traditional manner? Describe if any additional steps were necessary to improve the fidelity of the treatment when delivered remotely.

#### Item 12: HOW WELL (actual): if intervention adherence or fidelity was assessed, describe the extent to which the intervention was delivered as planned

Provide an assessment of how well the research team was able to track fidelity as well as any limitations or challenges that must be considered when assessing the adherence data of the telehealth intervention. Was there a way to determine if the instrument used to monitor adherence was valid in a remote environment?

### Summary

With the recent growth in remote delivery of NPTs, guidelines for reporting interventions can help facilitate replication of telehealth interventions used in clinical trials. To facilitate appraisal and implementation of telehealth interventions, we developed the TIDieR-Telehealth Intervention guideline to be used in conjunction with the original TIDieR checklist. This guideline recognizes that some components of telehealth interventions require additional explanation and elaboration, beyond the guidance of the original TIDieR checklist. Clear and reproducible descriptions of telehealth interventions are necessary for proper translation of research into clinical practice and for future validation and replication of studies.

### Strengths and limitations

The strength of this tool is that it was developed with feedback from a large collaboration of stakeholders involved in clinical trials focused on nonpharmacologic interventions (clinicians, trialists, other investigators). The input was provided by principal investigators using telehealth interventions in their own clinical trials, ranging from some who originally planned for the intervention to be delivered remotely to others that were required to pivot from in-person to a remote delivery due to the global pandemic affecting in-person care. While initially developed in the context of pragmatic trials studying nonpharmacologic approaches for pain management, the TIDieR-Telehealth checklist is applicable in clinical trials studying telehealth interventions for other clinical conditions. There are also some limitations to consider. It is primarily intended for use with interventions conducted with clinicians and patients and may not be relevant for non-clinical settings or interventions (i.e, community education). The perspectives driving the creation of this tool were primarily from that of clinicians and researchers that deliver NPTs for pain management. The majority of interventions delivered remotely, based on the definition we provided, are not likely to be medical procedures or pharmacological in nature. Regardless, the guide we present is relevant to the majority of telehealth interventions.

## Conclusions and recommendations

Even when results from clinical trials are promising, many end up having a minimal impact because the trial interventions cannot be replicated. The inability to implement interventions into clinical practice results in considerable waste of research resources. We developed the TIDieR-Telehealth to be used along with the original TIDieR checklist for additional guidance specific to reporting on telehealth interventions evaluated in clinical trials. We recommend that investigators studying telehealth interventions use this tool in both the planning and reporting of their trial interventions. Editors should consider encouraging this checklist in their guidelines for authors. Use of the TIDieR-Telehealth checklist will improve transparency, reproducibility, and the overall ability to implement research findings from remotely delivered interventions into practice.

## Data Availability

N/A.
